# Phytochemicals as novel agents for the induction of browning in white adipose tissue

**DOI:** 10.1186/s12986-016-0150-6

**Published:** 2016-12-03

**Authors:** Yusra Azhar, Ashish Parmar, Colette N. Miller, Janaiya S. Samuels, Srujana Rayalam

**Affiliations:** 1 Department of Pharmaceutical Sciences, School of Pharmacy, Philadelphia College of Osteopathic Medicine- GA Campus, 625 Old Peachtree Rd NW, Suwannee, GA 30024 USA; 2 Department of Foods and Nutrition, University of Georgia, Athens, GA USA

## Abstract

Obesity and its associated metabolic syndrome continue to be a health epidemic in westernized societies and is catching up in the developing world. Despite such increases, little headway has been made to reverse adverse weight gain in the global population. Few medical options exist for the treatment of obesity which points to the necessity for exploration of anti-obesity therapies including pharmaceutical and nutraceutical compounds. Defects in brown adipose tissue, a major energy dissipating organ, has been identified in the obese and is hypothesized to contribute to the overall metabolic deficit observed in obesity. Not surprisingly, considerable attention has been placed on the discovery of methods to activate brown adipose tissue. A variety of plant-derived, natural compounds have shown promise to regulate brown adipose tissue activity and enhance the lipolytic and catabolic potential of white adipose tissue. Through activation of the sympathetic nervous system, thyroid hormone signaling, and transcriptional regulation of metabolism, natural compounds such as capsaicin and resveratrol may provide a relatively safe and effective option to upregulate energy expenditure. Through utilizing the energy dissipating potential of such nutraceutical compounds, the possibility exists to provide a therapeutic solution to correct the energy imbalance that underlines obesity.

## Background

As the epidemic of obesity continues to grow, adipose tissue has increasingly become an area of focus for researchers. Adipose tissue plays an important role in the human body not just in terms of lipid accumulation but also in its endocrine functions. The expansion of white adipose tissue (WAT) and subsequent changes in circulating adipokines have been implicated in the pathogenesis of obesity [1]. Likewise, the perturbances in the activity of brown adipose tissue (BAT), the energy dissipating organ important for thermogenesis, also play an additional role in driving the obese-state. Because of this, activation of BAT has gained attention as a therapeutic target for obesity recently [2]. Many advancements have occurred in the area of brown fat technology, specifically relating to pathways in which BAT is functionally and physically different from WAT and various strategies that can be used to activate BAT. Discovery of brown adipocyte - like cells interspersed in WAT of human adults, termed beige or brite adipocytes [3], has further increased research investigating methods to activate these cells as an approach towards prevention and treatment of obesity. This review defines the current information on the function of BAT and mechanisms that drive its activation. Further, we will explore the current research on phytochemicals which have shown some promise as thermogenic agents or activators of BAT.

## White and brown fat adipogenesis

The life cycle of an adipocyte begins at the stage of a multipotent stem cell, which can differentiate into multiple cell types, including myoblasts and adipocytes. Expression of various transcriptional regulators such as peroxisome proliferator-activated receptor gamma (PPARγ) drives the differentiation of adipocytes. PPARγ is a hormone receptor specific to adipocytes that has been implicated as a key enhancer of adipogenesis. Activation of PPARγ occurs early in the preadipocyte life cycle and is regulated by a variety of lipids such as triglycerides, esters, and sterols [4]. While overexpressed C/EBPβ (CCAAT enhancer binding protein β) has the ability to promote adipogenesis in 3 T3-L1 preadipocytes, the knockout of this gene in conjunction with C/EBPδ results in a decreased number of adipocytes, leading to a reduced adipose tissue mass [5]. The knockout of C/EBPβ gene alone showed little effect on decreasing adipose tissue mass suggesting the redundancy of function by C/EBP family transcription factors. Nevertheless, early expression of C/EBPβ is required for adipogenesis and activates C/EBPα and PPARγ, the key transcription factors that work together to in turn activate a group of genes that promote adipogenesis (reviewed in [6]). The expression of these two genes leads them to positively cross activate one another and perpetuate the adipocyte lineage. Interestingly, PPARγ is not only involved in white adipogenesis but also plays a key role in the induction of brown adipocyte-specific genes (reviewed in [7]).

Unlike the majority of white adipocytes, the adipocytes found in BAT are derived from the Myf5 lineage and thus share a common precursor with skeletal muscle [8]. PR domain containing 16 (PRDM16) is a determining transcription factor for BAT development and overexpression of this protein results in browning of primary visceral preadipocytes [7]. PRDM16 promotes the induction of BAT genes by partnering with peroxisome proliferator-activated receptor γ coactivator (PGC-1) α and β. PGC-1α is a coactivator of PPARγ and it primarily controls mitochondrial biogenesis through the induction of uncoupling proteins such as uncoupling protein 1 (UCP1) [8]. Sirtuin-1 (SIRT1) is another important regulator of thermogenesis and its primary role is to deacetylate PPARγ [9]. Deacetylation of PPARγ is required to recruit PRDM16 which further leads to the induction of BAT genes and repression of WAT genes [7]. Association between these important transcription factors leads to the development and regulation of BAT function.

**Fig. 1:**
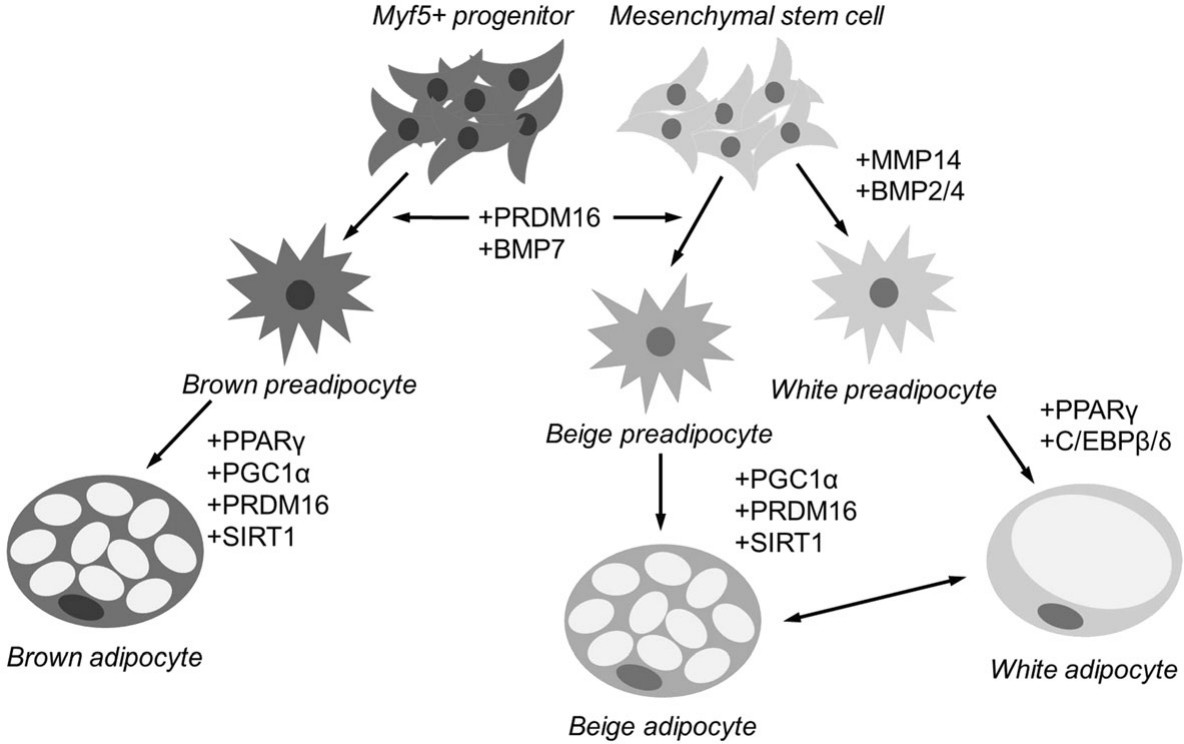
Developmental origins of *white*, *brown* and *beige* adipocytes. *Beige* adipocytes are derived from mesenchymal stem cells (Myf5^−^) and are closely related to *white* adipocytes, while *brown* adipocytes are derived from Myf5^+^ precursor cells and are closely related to muscle lineage

Not surprisingly, considerable cross-talk in the regulation between BAT and WAT exists where WAT-specific genes downregulate BAT activity. PRDM16 is required for beiging in WAT and the repression of genes that promote WAT development [7]. Mice that are deficient in adipose tissue-specific PGC-1α have dulled expression of thermogenic and mitochondrial genes in WAT [10]. Lastly, similar adipogenic factors that stimulate the differentiation of WAT such as PPARγ and C/EBPβ, also appear to be the drivers of BAT differentiation and hence are important regulators of adipogenesis for both cell types [11, 12] (Fig. 1).

## Thermogenesis

Thermogenesis is the process of converting chemical energy into heat. While shivering thermogenesis makes use of rapid muscular twitches to produce heat, BAT is specialized to generate heat in a process called non-shivering or adaptive thermogenesis [13]. BAT plays a pivotal role in protecting animals from hypothermia and is used during the periods of hibernation. It has long been known that BAT is present in newborns, but a number of recent studies conducted through the combined utilization of 18-FDG PET and CT show that human adults do have brown fat [14, 15] paving way to a new area in research relating to metabolic and obesity therapies [16]. The functional properties of BAT that makes it different from WAT mainly come from the lack of a large, unilocular lipid droplet and the presence of numerous mitochondria which allows for the production of energy. Mitochondria in brown adipocytes have low levels of ATP synthase and so cannot utilize the proton gradient of mitochondria to produce ATP. Instead, they employ UCP1 which uncouples cellular respiration and ATP synthesis, and thus results in the production of heat [17]. In vivo studies have shown that mice that lack the *Ucp1* gene preferentially express an obese phenotype [18]. These studies show the importance of BAT thermogenesis and its role in preventing obesity.

The sympathetic nervous system plays a significant role in the regulation of BAT thermogenesis. The release of catecholamines such as norepinephrine as a result of sympathetic stimulation from cold induction through the transient receptor potential (TRP) cation channels (members A1, M8, and V1) leads to the activation of the mitochondria in BAT which further leads to heat generation. The subsequent binding of norepinephrine to β-3 adrenergic receptors causes the secretion of free fatty acids from BAT which is the main energy source for UCP1 driven thermogenesis [19].

Thyroid hormone is an additional critical driver of the thermogenic response and brown adipose tissue activation. The conventional signaling cascade for thyroid hormone starts from the release of thyroxin (T_4_) from thyroid gland upon stimulation by the pituitary. Once released, T_4_ travels through the bloodstream to target tissues that express the necessary deiodinase (specifically DIO2) for the creation of triiodothyronine T3 [20]_._ Relative to other tissues, brown fat expresses a relatively large amount of deiodinase [21] and thus is reactive to changes in circulating T_4_ concentrations, in addition to the sympathetic activation that upregulates deiodinase expression [22]. The UCP1 promoter contains a transcriptional regulatory region for the thyroid hormone receptor β [23]. Thus, thyroid hormone can directly upregulate the expression of UCP1 and serves as a necessary regulator for both brown adipogenesis and thermogenesis. Further, the α-subtype of the thyroid hormone receptor also regulates the expression of the β-adrenergic receptors [24], thereby sensitizing brown fat to sympathetic activation. Secondarily, active T3 can be released from tissues and interact with additional cell types not believed previously to be regulated by thyroid hormone. Of most interest, T3 has demonstrated the ability to activate the ventral medial hypothalamus which serves as a central mediator of the sympathetic nervous system [25]. Through this mechanism, T3 appears to further regulate sympathetic activity and drive the activation of BAT in addition to direct transcriptional control of UCP1. It should be noted that the levels of T3 in BAT is influenced by DIO2 activity, which in turn is inhibited by T4 and activated by adrenergic stimulation. Thyroid hormone and the sympathetic nervous system are thus intimately tied, both co-regulating their respective responses and together drive the body’s response to cold [26].

## Phytochemicals in obesity

Natural, plant-derived compounds have made up the backbone of many of the synthetic drugs which are used today. The use of natural products for medical purposes dates back thousands of years; however their use in the discovery and development of modern drugs has only occurred since the early 19th century. Nearly 50% of drug approvals in the last 30 years came from compounds that were directly or indirectly derived from natural products [27]. The safety of these synthetic compounds however is hotly debated. Recent drug recalls and fatalities have led to resurgence in research on natural products because of their ease of use and efficacy. In particular, certain anti-obesity medications are removed from market owing to their adverse side effects [28]. In this context, natural products have been studied for their role in the regulation of adipocyte life cycle [29]. Phytochemicals can target different stages in the adipocyte life cycle by decreasing adipogenesis, inducing lipolysis, inducing adipocyte-apoptosis and by inducing transdifferentiation of white to brown-like adipocytes [30]. While the terms nutraceuticals, phytochemicals and bioactives are often used synonymously, it should be noted that phytochemicals are non-nutrient bioactive compounds found in fruits, vegetables and other parts of plants. Nutraceuticals on the other hand are broadly defined as food supplements that are used to improve health. This review focuses primarily on the effects of purified bioactive compounds rather than the plant extracts. In the coming sections, we discuss some of the phytochemicals that have shown promise as activators of BAT or have potential to act as thermogenic agents for future applications in the prevention and treatment of obesity and metabolic syndrome.

### Resveratrol

Resveratrol is a polyphenol found in a number of plants including the skin of grapes and several other types of berries. Numerous studies have indicated the anti-oxidant properties of this compound and the research around resveratrol continues to grow into other therapeutic uses such as cancer suppression and improving insulin sensitivity [31]. Studies on resveratrol’s effects on inflammation and thermogenesis have shown a decrease in the production of inducible nitric oxide synthase 2 (iNOS-2) and an increase in the markers of mitochondrial biogenesis contributing to an overall increase in energy expenditure [32].

In regards to obesity and adipogenesis, resveratrol decreases adipocyte differentiation and lipid accumulation via a decrease in the expression of key transcription factors involved in adipogenesis like PPARγ and C/EBPα [33]. Furthermore, SIRT1 in WAT is activated by resveratrol to promote the mobilization of fat from adipocytes [31, 33]. Unsurprisingly, resveratrol has shown the possibility to also regulate BAT activity. Alberdi et al. found elevated levels of UCP1 expression in the BAT and skeletal muscle of mice that were fed a diet supplemented with resveratrol [32]. Further, oral administration of resveratrol in mice also showed an increase in SIRT1 expression in WAT [34]. Authors proposed that the increased UCP1 expression seen in mice is due to stimulation of SIRT1 contributing to the improved energy efficiency and decreased fat mass. On the other hand, Um et al. reported that resveratrol fails to upregulate thermogenic proteins like PGC1α in adenosine monophosphate activated kinase (AMPK) null mice and AMPK null mice are resistant to the thermogenic effects induced by resveratrol [35]. Subsequent studies however revealed that SIRT1 plays a key role in potentiating resveratrol-induced activation of AMPK and improving mitochondrial function [36]. These findings suggest that resveratrol – induced increase in whole-body energy expenditure might be partly mediated by the induction of browning in WAT.

### Curcumin

Curcumin is a flavonoid found in turmeric, a spice popular in south Asian cuisine. Administration of curcumin has been shown to improve insulin sensitivity and increase weight loss in insulin-resistant obese mice [37].

Curcumin inhibits the early stages of adipogenesis in 3 T3-L1 adipocytes by lowering the expression of PPARγ and C/EBPα leading to a decrease in lipid accumulation [38]. Furthermore, curcumin-treated mice have lowered amount of free fatty acids, triglycerides, and improvement of insulin resistance and hyperglycemia suggesting its anti-diabetic potential [37]. Subsequently, curcumin has been shown to induce browning of 3 T3-L1 cells as indicated through increased expression of brown fat markers including PGC-1α, PPARγ, and UCP1 in dose dependent manner [39]. Further, T-box transcription factor 1 (TBX1), a beige specific marker, was significantly increased in 3 T3-L1 and primary white adipocytes following treatment with curcumin. Such findings have also been replicated in the mouse model where curcumin administration (50 or 100 mg/kg) resulted in the increased appearance of beige adipocytes in subcutaneous and inguinal WAT. Within this study, curcumin treatment resulted in the increased expression of many beige specific markers such as *Ucp1*, *Pgc1α*, *Dio2* and *Prdm16*. Cold tolerance tests conducted on mice showed that curcumin treated mice had increased body temperature compared to temperatures around 4 °C [40].

Additionally curcumin treatment stimulated the emergence of beige cells in inguinal and subcutaneous WAT but not epididymal WAT. The authors further postulated that the curcumin-induced browning of WAT is mediated by the upregulation of β3-adrenergic receptor expression and elevation of plasma levels of norepinephrine by curcumin [40]. Not surprisingly, curcumin appears to act through the transient receptor potential vanilloid receptor 1 (TRPV1) receptors located in the intestinal jejunum and thus may have downstream effects on both WAT and BAT through direct modulation of the sympathetic nervous system [41].

### Genistein

Soy isoflavones are phytoestrogens which have shown promise in lipid metabolism. A recent human clinical trial with isoflavone supplemented soy probiotic for 42 days, showed an improvement in the lipid profile of moderately hypercholesteremic men [42]. One major photochemical belonging to this group of soy isoflavones is genistein. Genistein is found primarily in soybeans and broad beans, which are harvested in parts of Western Asia and Europe. Effects of genistein on cancer prevention have been under investigation for a long time and these effects are attributed to the epigenetic effects of genistein. Genistein was shown to target all the epigenetic mechanisms like altering DNA methylation, and histone modifications that control the accessibility of genes of interest (reviewed in [43]).

Not only has genistein been described as a PPARγ agonist [44], but recent studies provide evidence that genistein has the potential to promote characteristics of beiging in WAT. High dose treatment of genistein (50–100 μM) on NIH3T3-L1 cells was shown to result in the increased expression of SIRT1 and its downstream partner, UCP1 [45]. Such an effect was also observed in primary culture whereas genistein increased mitochondrial biogenesis by upregulating PGC-1α [46]. Recent research however has shown confounding evidence on genistein’s anti-obesity effects. In 3 T3-L1 and human primary adipocytes, genistein has shown to inhibit adipogenesis at concentrations of 50 μM [47]. However, Zanella et al. found that using minimal doses (plasma concentration of 4 μM) in mice models, genistein promoted 3 T3-L1 adipogenesis rather than inhibiting it [48]. This evidence highlights the importance of dose range in the effect of phytochemicals on the cellular mechanisms of adipocyte differentiation. Genistein and its fellow isoflavone resveratrol have shown their ability to defend against metabolic syndrome by regulating lipid and glucose metabolism. Lastly, it is important to mention that the use of both resveratrol and genistein has shown to have a greater effect on adipogenesis and apoptosis of adipocytes rather than each of these compounds alone [49]. Thus, it is likely that the combination treatments of both genistein and resveratrol may lead to an even greater anti-obesity effect and activation of BAT which has not yet been explored.

### Guggulsterone

Guggulsterone (GS) is the bioactive gum resin derived from the bark of the *Commiphora mukul* tree predominantly found in India, Bangladesh and Pakistan. Cholesterol lowering effects of GS were first reported in hyperlipidemic rabbits [50] and since then, numerous animal and clinical studies have been conducted to demonstrate the effects of GS on lipid, cholesterol, and triglyceride levels [51]. However, there is a lack of reliability in several of the human studies that have attempted to explore and better understand the potential of GS due to flawed techniques [52]. In contrast, in vitro studies investigating the effects of GS on adiposity have found more success and clearly demonstrate i) inhibition of adipogenesis [53], ii) increase glucose uptake in insulin resistant conditions [54], iii) lipolytic effects in combination with other agents such as genistein [55] xanthohumol [56], and hormonal metabolite of vitamin D [57]. To date there is no scientific-based research that investigated the ability of GS to stimulate mitochondrial uncoupling and thus increase metabolic rate.

**Fig. 2:**
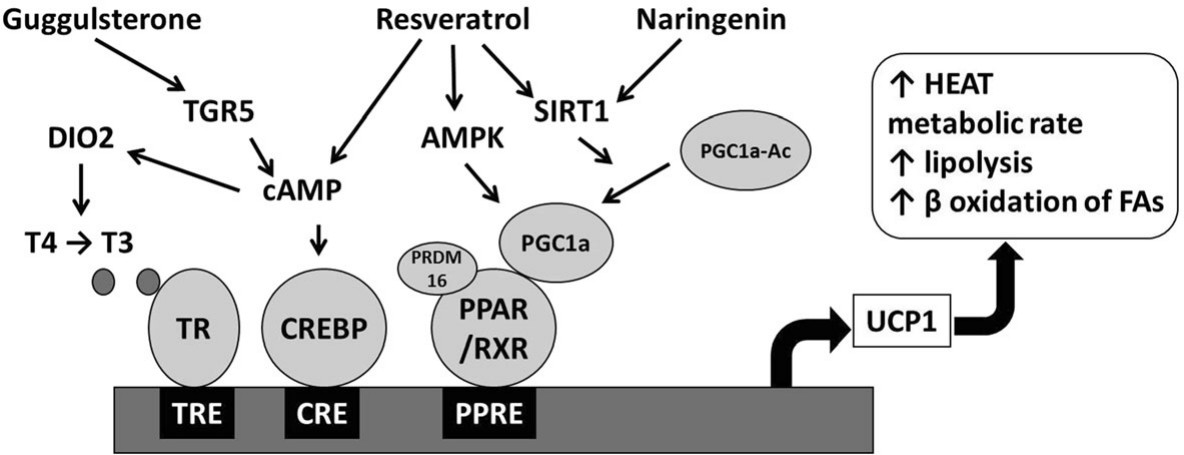
Model for the induction of browning by phytochemicals. Guggulsterone binds to TGR5 and increases the expression of DIO2 which in turn enhances T3 levels leading to the induction of beiging. Resveratrol is a sirtuin activator and enhances the levels of cAMP and also activates AMPK. SIRT1 mediates PGC1α deacetylation and AMPK activates PGC1α. PRDM16 co-activates PGC1α and PPARγ driving the upregulation of thermogenic genes. Likewise, naringenin also activates SIRT1 contributing to the induction of beiging. Abbreviations: AMPK (5’ adenosine monophosphate activated protein kinase), DIO2 (type 2 deiodinase), PGC-1α (PPARγ coactivator 1α), PPARγ (peroxisome proliferator-activated receptor γ), PRDM16 (PR domain containing protein 16), SIRT1 (sirtuin 1) and T3 (tri-iodothyronine)

GS is structurally similar to bile acids and has been identified as a selective bile acid receptor modulator [58]. Additionally, GS was also shown to exhibit thyroid stimulating activity [59], indicating potential for GS as a browning agent. Apart from interacting with farnesoid X receptor [58], a bile acid receptor, GS may also act as ligand for Takeda-G-protein-receptor-5 (TGR5), another bile acid receptor [59]. Bile acids have been implicated in weight control by reversing and preventing diet induced obesity [60]. TGR5 receptor is expressed in many of the gastrointestinal tract organs, lungs, mammary gland, uterus, skeletal muscle, macrophages and brown adipose tissue and mainly functions to increase intracellular adenosine monophosphate (AMP) [61]. Activation of TGR5 drives to increase cyclic AMP-dependent upregulation of DIO2 which is a response to sympathetic activation as well as increasing serum concentrations of thyroxine (T4) [59]. Deiodinase facilitates the conversion of T4 to 3,3′,5-triiodothyronine (T3), which is a critical component in UCP1 induction [62] (Fig. 2). GS has been shown to induce DIO2 expression in mature 3 T3-L1 adipocytes [63], further strengthening the hypothesis that browning effects of GS may, in part, be mediated via the activation of TGR5 signaling pathway.

### Xanthohumol

Hop plants or *Humulus lupulus* are more widely known for their usage in the beer brewing process but far less known are the historic uses of hops in traditional medicine. Xanthohumol, derived as the prenylated flavonoid of female inflorescences of the hop plant, has some promising anti-obesity effects. In addition to its in vitro effects on inhibiting adipogenesis [64] and causing apoptosis in mature adipocytes [65], xanthohumol also extends to in vivo effects where it is found to protect against diet induced obesity [66]. Xanthohumol increases energy expenditure which has been demonstrated in various cell types including white and brown preadipocytes, hepatocytes and myocytes [67]. Administration of xanthohumol increases oxygen consumption levels while ATP synthase was inhibited indicating the uncoupling of mitochondria.

The mechanism of xanthohumol’s effects on cellular metabolism is through increasing the production of reactive oxidative species, which leads to the activation of 5’ adenosine monophosphate-activated protein kinase AMPK and PGC1-α [67]. Interestingly, xanthohumol, like several other phytochemicals, exhibits hormesis effect, wherein low dose of xanthohumol increased uncoupling and oxygen consumption while high dose inhibited respiration in an ROS-dependent manner. Nevertheless, xanthohumol may ameliorate metabolic syndrome, at least in part, through mitochondrial uncoupling and stress response induction [67]. Xanthohumol also has an effect on bile acid generation which may lead to activation of bile acid G-protein coupled receptor TGR5 and downstream activation of T3 and ultimately UCP1 (Fig. 2). The uncoupling ability of xanthohumol can be attributed to its nonpolar nature and the ease of ability to which it can cross the plasma membrane and potentially activate transcription nuclear receptors which regulate metabolic genes [67].

While xanthohumol has demonstrated anti-obesity potential in rodent studies where dietary xanthohumol-rich hop extract significantly lowered body weight gain, its effects have been more related to WAT and very little attention has been placed on BAT [66]. Administering mature hop plants to mice was found to induce thermogenesis in brown adipocytes, which is demonstrated by increased expression of PPARγ and UCP1 [68]. Because xanthohumol has shown the potential to upregulate oxygen consumption rates and chemical uncoupling, it can be suggested that xanthohumol may be inducing such metabolic changes through systemic thyroid hormone signaling. Small, but significant increases in T4 binding globulin was seen following xanthohumol administration [69] and additionally, xanthohumol also upregulated iodide uptake by thyrocytes indicating a likely direct role in promoting thyroid hormone biosynthesis [70]. Future research should be placed on the metabolic impacts of xanthohumol and thyroid hormone signaling and further research is needed to definitively demonstrate xanthohumol’s potential on BAT activity

### Naringenin

Naringenin, a flavonoid found in citrus fruits such as grapefruits and oranges, has also been recognized as a bioactive compound with protective properties against adiposity. Significant evidence shows that naringenin prevents metabolic syndrome by inhibiting diet-induced dyslipidemia [71], lipogenesis [72] and adipogenesis [73].

Inflammation of the adipose tissue is one of the hallmarks of obesity. This inflammation is derived from an infiltration of macrophages in the adipose tissue [74]. The protective effects of naringenin were elucidated in one study where mice fed a high fat diet along with naringenin had decreased levels of macrophage infiltration and thus lower obesity-related adipose tissue inflammation [75]. Another study shows naringenin administered to rats in conjunction with cholesterol-rich diet reduced total cholesterol and triglyceride levels as well as increased antioxidant activity [76].

Furthermore, naringenin-fed mice also show an upregulation in gene expression of PPARα, a regulator of lipid catabolism [77]. In brown fat activators, PPARα is linked to fatty acid oxidation as a direct result of UCP1 induction and thermogenesis [78]. Further, PPARα-dependent induction of UCP1 is also found in WAT and is suggestive of the beiging potential of naringenin [79]. Preliminary studies conducted in our lab showed that naringenin at 25 and 50 μM concentration induced a dose-dependent increase in the expression of UCP1 and SIRT1 in primary human omental adipocytes. These preliminary experiments suggest a possible potential for naringenin as a thermogenic agent with therapeutic applications in obesity and metabolic syndrome.

### Quercetin

Another flavonoid found to have beneficial anti-obesity effects is quercetin (3,3′,4′,5,7-pentahydroxyflavone). Commonly found in high concentrations in apples, broccoli, berries, onions, leafy vegetables and asparagus, quercetin is a polyphenol that has significant data showing its beneficial effects on cardiovascular system and lipid homeostasis [80]. Quercetin supplementation in high fat diet-induced obese mice protects against diet-induced obesity by increasing energy expenditure and inflammation [81]. Other studies demonstrating the protective effects of quercetin supplementation in mice fed high fat diet found lower body weight gain, triglycerides, and plasma cholesterol levels as a result of improved regulation of metabolic genes [82]. Quercetin also improved metabolic conditions in obese mice, as demonstrated by improved dyslipidemic state [83]. In vitro studies of quercetin rich extract showed inhibition of adipogenesis, decreased lipid accumulation and apoptosis of mature white adipocytes [84].

Dietary quercetin has also shown the ability to increase the expression of UCP1 and thus thermogenesis in mice fed with quercetin-enriched diet [85]. In this study, quercetin was shown to inhibit polarization of bone marrow-derived macrophages towards pro-inflammatory M1 lineage through an AMPK/SIRT1-mediated mechanism. Given the well-established role of SIRT1 and AMPK in energy expenditure [86] it is likely that quercetin has the potential in induce browning of WAT. Although in vitro and in vivo studies provide significant data in support of quercetin related response to adiposity and obesity, the direct effects of quercetin on white adipocyte transdifferentiation needs to be further researched.

### Capsinoids

Capsaicinoids are a group of phytochemicals including, but not limited to, capsaicins and capsinoids. The capsaicinoid family consists of capsaicin, dihydrocapsaicin, nordihydrocapsaicin and others. There has been extensive research showing that capsaicin has anti-obesity, anti-diabetic, and anti-inflammatory characteristics. Recent studies also indicate that capsaicin acts by activating the sympathetic system to induce BAT thermogenesis and reduce fat accumulation [87].

Administration of capsaicin in mice has shown to induce thermogenesis via the activation of BAT [88]. This is evidenced by the increase in markers related to mitochondrial biogenesis such as PPARγ, PGC-1α and UCP1 [88]. Capsaicin also induces the development of beige adipocytes at an early stage of adipogenesis [88]. The effect of capsaicin/capsinoid treatment has been compared to that of chronic cold exposure, in which sympathetic stimulation results in the activation of BAT [87]. Similarly to the aforementioned curcumin, capsaicin binds to the intestinal transient receptor potential vanilloid 1(TRPV1) receptor, also referred to as the capsaicin receptor, thereby launching the sympathetic response observed with treatment [89]. Surprisingly, capsaicin has also been proven to be harmful to humans and these harmful effects are mediated, in part, by the capsaicin receptor, TRPV1 [90–92].

Capsinoids are capsaicin analogs, similar in function to capsaicins, but are far less pungent thus, less toxic, and physiologically compromising to humans. The low pungency characteristic of the naturally occurring ‘CH-19 Sweet’ pepper makes them edible in comparison to capsaicinoids and therefore, are an attractive target for anti-obesity therapy.

It has been demonstrated that capsinoids decrease fat accumulation in adipocytes both in vitro and in vivo in mice [93]. Acute administration of capsinoids augments energy expenditure, sympathetic nervous system activation, and thermogenesis with comparable efficacy to capsaicins. Capsinoids are TRPV1 agonists and increased energy expenditure and fat oxidation is dependent on TRPV1, much like capsaicin, but the capsinoid sensory receptor is found in the gastrointestinal tract while capsaicin’s sensory receptor is located on the tongue. The difference in the location of sensory neurons and capsinoid’s subtle pungency decreases its likelihood for hyperalgesia effects in comparison to capsaicin [94].

As an inducer of the browning of WAT, a diet supplemented with capsinoids fed to mice kept at 17 °C for 8 weeks, significantly increased energy expenditure, but not at 25 °C. This was confirmed by increased BAT and beige specific gene markers such as *Ucp1, Pgc1α, Cidea, Cd137,* and *Tmem26*, in inguinal WAT, respectively. It has been shown that capsinoids upregulated the expression of the PRDM16 protein in inguinal WAT under ambient and mildly cold temperatures upon β-adrenergic stimulation [95]. Yoneshiro et al. demonstrated that acute administration of capsinoids increased energy expenditure in BAT-positive subjects, but not in subjects without metabolically active BAT, under cold exposure [96]. Capsinoids seem to be promising in that they are accompanied with fewer side effects than capsaicins but there are conflicting studies of their potential as browning agents. Therefore, further research needs to establish its role in the browning of WAT and the associated underlying molecular mechanisms.

### Cinnamaldehyde

Cinnamaldehyde is a pungent spice extracted from the plant cassia and is the most abundant phytochemical in cinnamon [97]. Used since the medieval times for medicinal purposes, cinnamaldehyde has now been identified to have multiple therapeutic uses such as anti-diabetic, anti-arthritic, anti-inflammatory, anti-microbial, and anti-cancer effects [98]. Cinnamaldehyde activates TRP cation channels, similar to capsaicinoids. More specifically, cinnamaldehyde activates the cold-gated ion channel, transient receptor potential Ankyrin subtype 1, TRPA1 [99]. It has been shown that the cinnamaldehyde acts as an agonist to TRPA1 and upregulates adrenaline secretion in rats [100]. This adrenaline secretion stimulation by cinnamaldehyde could explain the induction of thermogenesis and inhibition of heat diffusion in mice [101].

Cinnamon decreased lipid accumulation in vitro in 3T3-L1 preadipocytes and the expression of adipogenic transcription factors PPARγ, C/EBPα and SREBP-1c during adipocyte differentiation [102]. In a dose-dependent manner, cinnamaldehyde also decreased visceral fat deposition, partly mediated by the activation of interscapular BAT, as evidenced by the upregulation of UCP1 expression levels, in high fat and high sucrose diet-fed mice [97]. Taken together, this data suggests the potential of cinnamaldehyde to act as a browning agent and exert its anti-obesity effects with future research.

### Fucoxanthin

Fucoxanthin, extracted from edible brown alga, is a carotenoid known to have anti-carcinogenic, anti-inflammatory, anti-diabetic and apoptotic effects in metastatic cells. Fucoxanthin has been shown to ameliorate the progression of obesity in vitro and in vivo in mice and human models [103]. Fucoxanthin reduced lipid accumulation accompanied by a decrease in PPARγ expression in 3T3-L1 adipocytes [104]. Kang et al. demonstrated that fucoxanthin stimulated 3T3-L1 adipogenesis at an early stage mediated by an increase in the expression of PPARγ and C/EBPα and the adipocyte differentiation marker, aP2. However, fucoxanthin significantly downregulated the adipogenesis of 3T3-L1 adipocytes at the intermediate and late stages of differentiation, and the expression of PPARγ, C/EBPα and SREBP1c transcription factors [105].

Maeda et al. investigated the potential anti-obesity and anti-diabetic effects of fucoxanthin supplemented diets in rodent models. Results from these studies suggested that fucoxanthin significantly lowered WAT weight gain in mice with high fat diet-induced obesity, as well as mRNA levels of leptin in WAT. Further, fucoxanthin stabilized blood glucose and insulin levels and downregulated monocyte chemoattractant protein-1, MCP-1, expression in WAT of diet-induced obese mice. MCP-1, a protein secreted from adipose tissues, stimulates macrophage accumulation and the production of pro-inflammatory mediators. Finally, β3-adrenergic receptor, Adrb3, mRNA expression levels were upregulated in WAT of mice maintained on fucoxanthin high fat diets. As discussed earlier, Adrb3, expressed in both BAT and WAT, is suggested to play a role in lipolysis and thermogenesis [106].

Fucoxanthin-fed obese mice experienced a decrease in WAT weight as well as a significant upregulation in the expression of UCP1 protein and mRNA in WAT, resulting in energy expenditure in the form of heat and fatty acid oxidation in WAT [107]. This increase in UCP1 expression was nearly diminished in WAT in mice maintained on a control diet. In another study of Maeda and his colleagues, fucoxanthin significantly decreased the body weight of mice on high fat diets [108]. Overall, these studies suggest that fucoxanthin may have promising anti-diabetic and anti-obesity effects and deserve more research focus, primarily in human subjects.

## Conclusions

**Fig. 3:**
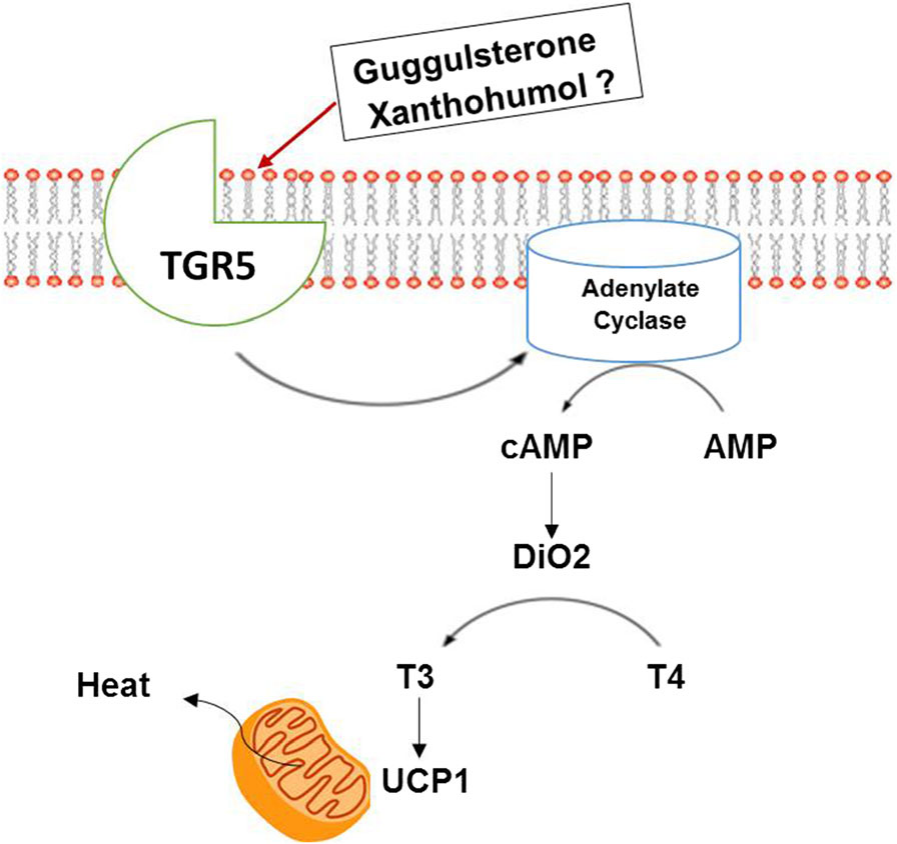
Possible TGR5-medaited effects of guggulsterone and xanthohumol. The structural similarity of guggulsterone and xanthohumol to bile acids allows them to bind to bile acid receptor TGR5 which induces cAMP mediated upregulation of DIO2. DIO2 converts T4 to biologically active T3, which in turn induces UCP1 and increases thermogenic activity of mitochondria. Abbreviations: cAMP (cyclic adenosine monophosphate), DIO2 (type 2 deiodinase), UCP1 (uncoupling protein 1)

Natural compounds have clear stimulatory effects on energy metabolism through direct actions on TRP channels and subsequent sympathetic signaling, intracellular regulation of the SIRT1-PRDM16 pathway and through modulation of thyroid hormone (Fig. 3). Through these mechanisms, natural compounds can promote chemical uncoupling and energy dissipation in brown adipose tissue that may be able to counteract the loss of function of brown fat seen in obesity. While safety and efficacy will always be in question with nutraceuticals, the specific compounds described herein have been safely used for hundreds of years without major adverse events that render them unsafe for use. Future research is needed to more appropriately answer the questions on efficacy, as some compounds which have the potential to stimulate brown adipose tissue have not been thoroughly investigated alone or in combination with other natural products that may act synergistically. Similarly, few compounds have been used in large, randomized clinical control trials to definitively answer their potential anti-obesity effects. Despite this, the mechanistic data in both cell and rodent models show promise that natural, plant-derived compounds do contain the capacity to promote a beneficial metabolic profile.

## Abbreviations

AMPK: Adenosine monophosphate-activated protein kinase
BAT: Brown adipose tissue
C/EBP: CCAAT enhancer binding protein
DIO2: Type 2 deiodinase
GS: Guggulsterone
iNOS: Inducible nitric oxide synthase
PGC1a: Peroxisome proliferator-activated receptor γ coactivator
PPAR: Peroxisome proliferator-activated receptor
PRDM16: PR domain containing 16
SIRT1: Sirtuin 1
SREBP1c: Sterol regulatory element-binding protein 1c
T3: Tri-iodothyronine
T4: Tetra-iodothyronine
TBX1: T-box transcription factor 1
TGR5: Takeda G-protein coupled receptor 5
TRP: Transient receptor potential
UCP1: Uncoupling protein 1
WAT: White adipose tissue
